# Approach to identifying research gaps on vector-borne and other infectious diseases of poverty in urban settings: scoping review protocol from the VERDAS consortium and reflections on the project’s implementation

**DOI:** 10.1186/s40249-018-0479-3

**Published:** 2018-09-03

**Authors:** Stéphanie Degroote, Clara Bermudez-Tamayo, Valéry Ridde

**Affiliations:** 1 University of Montreal Public Health Research Institute (IRSPUM), Montreal, Quebec, Canada; 2 Andalusian School of Public Health, Granada, Spain; 3 Biomedical Research Network Centre for Epidemiology and Public Health (CIBERESP), Madrid, Spain; 4 French Institute for Research on Sustainable Development (IRD), Paris Descartes University (CEPED), Paris Sorbonne Cities University, Erl Inserm Sagesud, Paris, France

**Keywords:** Protocol, Scoping review, eDelphi, VERDAS consortium

## Abstract

**Background:**

This paper presents the overall approach undertaken by the “VEctor boRne DiseAses Scoping reviews” (VERDAS) consortium in response to a call issued by the Vectors, Environment and Society unit of the Special Programme for Research and Training in Tropical Diseases hosted by the World Health Organization. The aim of the project was to undertake a broad knowledge synthesis and identify knowledge gaps regarding the control and prevention of vector-borne diseases in urban settings.

**Methods:**

The consortium consists of 14 researchers, 13 research assistants, and one research coordinator from seven different institutions in Canada, Colombia, Brazil, France, Spain, and Burkina Faso. A six-step protocol was developed for the scoping reviews undertaken by the consortium, based on the framework developed by Arksey and O’Malley and improved by Levac et al. In the first step, six topics were identified through an international eDelphi consultation. In the next four steps, the scoping reviews were conducted. The sixth step was the VERDAS workshop held in Colombia in March 2017.

**Discussion:**

In this article, we discuss several methodological issues encountered and share our reflections on this work. We believe this protocol provides a strong example of an exhaustive and rigorous process for performing broad knowledge synthesis for any given topic and should be considered for future research initiatives and donor agendas in multiple fields to highlight research needs scientifically.

**Electronic supplementary material:**

The online version of this article (10.1186/s40249-018-0479-3) contains supplementary material, which is available to authorized users.

## Multilingual abstract

Please see Additional file 1 for translation of the abstract into the five official working languages of the United Nations.

## Background

More than 50% of the world’s population currently lives in cities and by 2050, around 70% of the global population are projected to be living in urban settings, mainly in low-and middle-income countries (LMICs) [1]. Mobility, poverty, inequality, and climate change are some of the social and environmental drivers of health risks in urban settings, including vector-borne diseases (VBDs) [2], which pose imminent public health challenges that require significant intersectoral policy and action. In that context, a broad knowledge synthesis was needed to guide future research.

This paper presents the overall approach undertaken by the VERDAS (“VEctor boRne DiseAses Scoping reviews”) consortium in response to a call issued by the Vectors, Environment and Society (VES) unit of the Special Programme for Research and Training in Tropical Diseases (TDR) hosted by the World Health Organization (WHO). The aim was to to perform a broad knowledge synthesis and identify knowledge gaps regarding the control and prevention of vector-borne diseases in urban settings.

There are many types of review methods (e.g. systematic reviews, rapid reviews, critical reviews, literature reviews, mixed-method reviews, state-of-the-art reviews, scoping reviews, etc.), and selecting from them requires careful consideration of the research questions and goals [3]. The different types present specific strengths and limitations and are suited for different purposes. The TDR call to which we responded specified the type of review desired as “state-of-the-art scoping reviews,” which are actually two different types of reviews. The first, state-of-the-art reviews, tend to address current matters and offer new perspectives for further research [4]. The second, scoping reviews, generally offer a preliminary assessment of the size and scope of available research literature and identify the nature and extent of research evidence [5]. The analysis in a scoping review is thus very exploratory [4], whereas state-of-the-art analysis describes current knowledge with a view to setting priorities for future investigation.

We decided to structure our approach based on the theoretical framework for scoping reviews developed by Arksey and O’Malley, [4, 5] and improved in subsequent publications [6, 7], combined with more in-depth analysis within the framework of a state-of-the-art review. The scoping review is a recent type of review that is becoming increasingly popular [8] but is still evolving, and as such there is some variability and lack of consensus on terminology, definition, methodological conduct, and reporting. The six-step framework for scoping reviews upon which we based our approach involves: 1) identifying the research question; 2) searching for relevant studies; 3) selecting studies; 4) charting the data; 5) collating, summarizing, and reporting results; and 6) consulting with stakeholders to inform or validate study findings.

Developing the present protocol was essential to the successful coordination of the consortium. To ensure consistency among teams, we established validation steps and systematic procedures, even when these might be contradictory to the scoping review approach. As the work advanced, we realized this may have led us beyond the traditional scoping reviews framework and towards systematic mixed-method reviews, which we will discuss further in the last section of this paper.

## Methods: a six-step protocol

### STEP 1: defining the research question

This first step consisted of an eDelphi consultation conducted from March to June 2016. By the end of the consultation we had obtained the six research questions for the VERDAS scoping reviews. All research topics suggested during the consultation are available in Additional file 2.

#### Assembling the panel of experts

Worldwide experts on vector-borne diseases (VBDs) were identified mainly through professional networking and snowball technique sampling (getting individuals to refer those they know, these individuals in turn refer those they know and so on) [9]. We conducted Internet searches and consulted publicly available lists of participants at scientific events. Our aim was to recruit at least 50 experts to ensure sufficient diversity, both geographic and professional. As such, we invited 201 relevant experts, on the assumption that a 25% positive response rate was achievable based on previous publications [10–12]. In fact, we had a positive response rate of 52% and were able to assemble a panel of 82 experts; we also received 22 refusals. Table 1 describes the panel in detail. Experts were invited by email, and all correspondence and surveys, designed and disseminated with the online free platform SurveyMonkey (www.surveymonkey.com) were conducted in English, French, and Spanish to enhance recruitment potential. In the invitations for each round, panelists received: a specific ID number, to anonymize their participation; a back-up copy of the survey in Word format (Microsoft Corporation, Redmond, WA, USA), in case of technical difficulties; and an anonymized summary of responses to the previous round. Invitations to the three consultation rounds were sent to all panelists regardless of whether they had participated in the previous rounds of consultation.

**Table 1:** Description of the eDelphi panel

	Total	Africa	Americas	Europe	Asia
Decision-makers	39	17	11	2	9
Ministry of Health: 12		Benin: 7	Argentina: 2	Switzerland: 1	Cambodia: 1
International level: 3		Burkina Faso: 2	Brazil: 1	United Kingdom: 1	Lao PDR: 1
National governance: 18		Cameroon: 1	Costa Rica: 1		Malaysia: 1
Regional governance: 5		Ethiopia: 1	Dominica: 1		Myanmar: 2
		Nigeria: 1	Haiti: 1		Republic of Korea: 1
		Rwanda: 2	Canada: 1		
		Tanzania: 1	Nicaragua: 1		Singapore: 1
		Uganda: 1	Venezuela: 1		Thailand: 1
		Ivory Coast: 1	USA: 2		Vietnam: 1
Private industries	3		1	2	
Vector control industry: 3			USA: 1	France: 1	
				Switzerland: 1	
Researchers	41	13	12	15	1
Anthropology: 3		Burkina Faso: 3	Brazil: 1	Belgium: 1	Myanmar: 1
Entomology: 7		Ghana: 1	Canada: 2	Germany: 2	
Epidemiology: 8		Gabon: 1	Cuba: 4	France: 6	
Geography: 1		Ivory Coast: 4	Honduras: 1	Norway: 1	
Public health: 11		Kenya: 1	Colombia: 1	Switzerland: 3	
Sociology: 1		Mali: 1	Mexico: 2	United Kingdom: 2	
Statistics: 1		Nigeria: 1	Peru: 1		
Virology: 9		Senegal: 1			

#### First round: suggestions

The objective was to compile an exhaustive list of all potential topics to consider for knowledge synthesis. To delineate the exercise and stimulate reflection, a list of 10 pre-identified topics was suggested, and panelists were asked to propose additional questions or topics. The pre-identified topics were the seven suggested by TDR in the call for projects and three added by our consortium in our response to the call. Ultimately none of these topics was selected (see Additional file 2). Panelists had 2 weeks to respond to the online survey, and we received 52 completed surveys (63% participation rate) with 161 additional research topics suggested.

We reworded all topics to be more compliant with our project (added urban context, removed a specific disease and replaced it with the general term “VBDs”). We grouped together topics on the same subject and excluded 11 that were either irrelevant for our goals (e.g. mobilization of innovative funding against poverty), or excessively broad, such that they were not suitable as a topic for a single review (e.g. research on VBD identification and management). In the end, we obtained a total of 75 topics in addition to the ten original topics.

#### Second round: ranking

The 85 topics were sorted into eight categories: Society (7 topics); Healthcare (12); Interventions for vector control (20); Surveillance, prevention and risk communication (15); Economics (6); Ecology and geography (10); Politics (8); and Methodology (7). The order of categories was randomized during the survey design and was the same for all participants. Topics within each category were automatically randomized at the opening of the survey link and thus presented in a different order for each participant. Panelists were invited to rate each topic in each category as follows: 1 = to eliminate; 2 = negligible; 3 = possible; 4 = desirable; 5 = essential/top priority.

Panelists had 2 weeks to respond to the online survey; we received 48 completed surveys. Despite the survey length and time needed to perform this ranking exercise, the participation rate was very satisfactory at 58%.

#### Third round: final selection of top priority topics

Topics were newly suggested to the panelists, presented in three categories (as before, topics were automatically randomized at the opening of the survey link within each category):

1. One topic with the highest ranking, i.e., the only one rated 4 or 5 by more than 85% of the panelists in round 2. Panelists were asked whether they had strong objections to including it in the final list.
2. Nineteen topics with medium ranking, rated 4 or 5 by more than 60% of the panelists in round 2. Panelists were asked to rate each of them again. The rating system was the same as before: 1 = to eliminate; 2 = negligible; 3 = possible; 4 = desirable; 5 = essential/top priority.
3. Sixty-five topics with the lowest rankings, rated 4 or 5 by less than 60% of the panelists. Panelists were asked whether they had strong objections to excluding them from the final list.

Panelists had 2 weeks to respond, and we received 49 completed surveys (59% participation rate). The six topics with the highest rankings are presenting in Table 2.

**Table 2:** The six final topics for scoping reviews chosen by the panel for the VERDAS consortium

	TOPIC	% panelists rating 4 or 5	Mean score
1	Development and implementation in urban areas of low-cost, simple and/or rapid diagnostic technologies for vector-borne and other infectious diseases of poverty	> 85%^a^	4.29 ± 0.87
2	Development of effective surveillance systems for vector-borne diseases in urban settings and translation of data into action	88%	4.29 ± 0.91
3	Evaluation of impact, cost-effectiveness and sustainability of integrated vector management in urban settings to prevent vector-borne diseases	79%	4.08 ± 0.71
4	Transmission dynamics, vectorial capacity and coinfections: impacts on the epidemiology of vector-borne diseases in urban areas	76%	3.90 ± 0.92
5	Evaluation of effectiveness of containment measures of emerging and re-emerging vector-borne and other infectious diseases of poverty	71%	4.00 ± 1.02
6	Housing, hygiene, sanitation and water, waste and infrastructures management in vector-borne diseases prevention in urban areas	63%	3.88 ± 1.07

^a^Score obtained in Round 2 while other topics score are from Round 3. This topic was automatically included in Round 3 with no objection during round 3. The consensus about this topic was obtained sooner than other topics which explain the order as number one even though topic number 2 has an actual higher final score

### STEP 2: identifying relevant studies

For each topic, three to five key concepts were defined using simple, short sentences associated with as many keywords as possible, some broader, some narrower. Two concepts were common to all reviews: vector-borne diseases and urban areas; as such, standardized lists of keywords were used across the consortium. For example, for the key concept of *vector-borne diseases*, we used keywords such as: *vector-borne diseases*; *neglected tropical diseases*; *tropical infectious diseases; malaria; dengue*; *leishmaniasis;* etc. Once the search strategy was finalized, the same exhaustive list of keywords was used to search all databases in titles and abstracts of papers. All complete search strategies for the VERDAS reviews are provided in appendices in each article.

We defined which databases to search based on each team’s access and the databases’ relevance for the topic (see Table 3). The following databases were used across all teams: PubMed, Embase, and Global Health for scientific literature, and Wholis and OpenGrey for grey literature. Based on the keywords list, we defined appropriate descriptors for each scientific database, as they vary from one database to another (e.g. MeSH terms for PubMed; EMTREE for Embase, etc.).

**Table 3:** Databases used by the VERDAS consortium and their main specificities or limitations

Databases used	Field of research / Interest	Specificities or possible limitations
CINAHL	Health / Nursing	Most records are citations only
Cochrane Library	Health / Biomedical	Only systematic reviews
EconLit	Economics	Cover all economics-related fields of research
Embase	Health / Biomedical	Strong focus on drug and pharmaceutical research
Global Health	Public Health	Strong focus on public health practice internationally
LILACS	Health / Biomedical	Only Spanish-speaking literature
Medline	Health / Biomedical	Very similar to PubMed, updated weekly
OpenGrey	Multidisciplinary grey literature	Strong focus on literature produced in Europe
PubMed	Health / Biomedical	Very similar to Medline, updated daily
Scopus	Multidisciplinary	World’s largest abstract and citation database, may be difficult to find full text
GreyLit	Health grey literature	No longer updated since January 2017
Google Scholar	Multidisciplinary	No advanced search possible, nor saving searches, nor exporting results; same search may lead to different results
Web of Science	Multidisciplinary	Based on citations, very broad; full text may be difficult to find
WHOLIS	WHO databases	Only WHO documentation; impossible to save searches and export results

All search strategies were reviewed several times and were launched only after validation by a specialized librarian and the consortium coordinator to ensure replicability and standardization among the consortium. Because database searches may not always go as planned, a second validation of all search histories was performed by the research coordinator and the librarian. The reference lists of all included articles were also cross-checked for relevant studies.

References retrieved were saved in either Zotero (www.zotero.org) or Mendeley (www.mendeley.com) reference manager software. Initially we opted to use Zotero for all teams to facilitate coordination and problem-solving, but some teams that retrieved very large numbers of references experienced technical difficulties with that software (slowness and abrupt shutdown), so we shifted towards Mendeley. We chose these two programs to be able to share complete libraries among all contributors and the consortium coordinator, for purposes of transparency and standardization

### STEP 3: selecting the studies

After all duplicates were removed, each team performed a pilot round of screening with 20 randomly selected references. Two contributors screened titles and abstract, labelled (included or excluded) each of the 20 citations independently, and provided an explanation for their decision. Then the two contributors met to discuss their choices, with a view to reaching a common understanding of the criteria and how they were to be applied. Because the criteria were determined and/or adjusted post hoc, based on increasing familiarity with the literature, the pilot round was fundamental. The final criteria were reviewed and validated by the coordinator of the consortium to ensure standardization among all teams.

The validated criteria were applied to all references by two independent reviewers based on titles and abstracts, and reasons for exclusion were recorded for each reference. When the reviewers did not reach consensus, a third independent reviewer was called in to resolve the disagreement (the team leader when she/he was not involved as reviewer, or the consortium coordinator). Any new contributor to the selection process first performed a pilot screening test, validated by the team leader, to ensure a common understanding of the criteria and their application. Given that an abstract cannot entirely reflect an article’s content, when a doubt persisted or information was lacking, the reference was included so that the full text would be screened. When the selection based on abstracts was completed, all references labelled “included” were kept for the subsequent step, and those labelled “excluded” were removed from the database. The same selection process was performed for the full text screening. All reasons for exclusion were detailed and then compiled in the Preferred Reporting Items for Systematic Reviews and Meta-Analyses (PRISMA) Flow Diagram.

Reference lists in all included articles were then manually checked for potential additional studies. After this, no further study was included in the review

### STEP 4: charting the data and quality assessment

#### Validation and adaptation of the data extraction tool

A grid was created beforehand using Excel (Microsoft Corporation, Richmond, WA, USA) that combined several validated tools used to collect macro- and micro-data from the selected literature, namely the Mixed Method Appraisal Tool (MMAT), Template for Intervention Description and Replication (TIDieR) and Analysis of the transferability and support to adaptation of health promotion interventions (ASTAIRE) tools. Each team performed a pilot round to ensure their understanding of the grid and its application and, as before, standardization among the consortium. Five studies were selected randomly for data extraction by two independent contributors, i.e., the research assistant (or the scoping leader) mainly involved in data extraction and the team leader (if appropriate) or the consortium coordinator. The number was decided intuitively to have enough data to fully test the grid, and, when necessary, more studies were selected to have a more diverse panel of studies. The grids were compared, and we arbitrarily settled on a disagreement threshold of 15% between the two contributors to validate the grid. To calculate the percentage of disagreement, row by row (i.e., study by study), we compared each cell and scored one point of disagreement when the cells did not contain the same data, or when a cell was completed in one grid and empty in the other. We then applied, for each row, the formula: [(points of disagreement × 100) / total number of cells in the row] = percentage of disagreement. If disagreement was under 15% for every study, the tool was validated and extraction proceeded. If one or more studies presented more than 15% disagreement, the data extraction tool required revision. The contributors discussed their challenges with the tool and the possible need to add variables to the grid. They also endeavoured to ensure they had the same understanding of each of the tools’ variables. After this pilot round, a second round of validation (or more if needed) was performed following the same protocol until they were below the 15% disagreement threshold. For all groups, only two rounds were sufficient to validate the grid. Even though the contributors may have found this step to be fastidious, it was essential to ensure the future usefulness of the data extraction tool for data synthesis and analysis. Any contributors subsequently added to a team and involved in data extraction underwent data extraction training based on the protocol, which was then validated by the team leader.

#### Quality assessment with the mixed methods appraisal tool

We used the MMAT checklist for quality assessment of all studies included. It is designed for the appraisal stage of complex systematic literature reviews that include qualitative, quantitative, and mixed-methods studies. The items of the tool were included in the second section of the data extraction grid.

#### Macro-data extraction with the template for intervention description and replication

The third section of the data extraction grid was based on the TIDieR checklist (Template for Intervention Description and Replication). A health-related intervention is defined by WHO as “an activity or set of activities aimed at modifying a process, course of action or sequence of events in order to change one or several of their characteristics such as performance of expected outcome” [13]. This definition encompasses a very wide variety of studies, such as studies on medication, health services, programs related to health habits, etc. The TIDieR was developed to help researchers report health interventions appropriately, as its authors observed that, in many reports, interventions are insufficiently or poorly described. Because the TIDieR categories are easily understandable and therefore easily transferable to other types of studies, we also used this checklist to extract data from non-intervention types of studies.

#### Micro-data extraction with ASTAIRE (a tool to analyze the transferability of health promotion interventions)

The fourth and last section of the data extraction grid was based on the ASTAIRE tool, a very detailed tool for describing intervention studies’ contexts. It is used to analyze the transferability of interventions in order to support their design, planning, and adaptation to new settings. An intervention’s transferability is defined as “the extent to which the measured effectiveness of an applicable intervention could be achieved in another setting” [14]. Given this tool’s level of detail and length, it was only possible to use it for reviews with a majority of intervention studies.

### STEP 5: collating, summarizing, and reporting the data

Our goal was to analyze the data extracted, report them as clearly as possible, and mainly apply meaning to the results. First, the following questions were provided to guide the writing of the manuscripts:

- What is known, what is currently done (where and in what context) and within which policy frameworks?
- What mechanisms trigger which outcomes?
- What proven principles or lessons could inform research, practice, and policy?
- What are critical knowledge gaps or research questions needing to be addressed in the future?
- What should be better known to guide action and policy?
- What are critical gaps in practice and policy based on available knowledge?

We also developed a scoping review template to help each team begin drafting its manuscript (Additional file 3). During this step, several rounds of internal reviews were performed, both within the research team and within the consortium. We observed that it was more effective to initiate reviews as soon as possible. Developing an outline of the manuscript and sharing it with all co-authors helped align expectations and orient the manuscript, and it was more difficult to reorient a manuscript when it was already very far advanced.

All consortium members committed themselves to a transparent distribution of authorship. We used the present protocol to develop a table for each step, in which contributors entered their name and time spent on their tasks. By the end, each team had a detailed table showing all contributors and time invested. Each team held an open discussion among all contributors to discuss the distribution and order of authorship.

### STEP 6: consultation of experts

While this step was considered optional in the original scoping review framework, [5] it turned out to be a key asset for finalizing the reviews. From the beginning of the research project, an international workshop was planned to bring together 14 VERDAS members (i.e., all team leaders, the principal investigator, the research coordinator, the knowledge translation expert, and some available research assistants) and eight decision-makers from policy-based institutions. When all teams had a preliminary draft ready to share presenting all major findings, we held a two-day workshop in Cali, Colombia, at the University of the valley. Objectives were to: 1) exchange knowledge to supplement the reviews; 2) identify research priorities based on findings; and 3) initiate the knowledge transfer strategy. Research priorities were prioritized by means of a concept mapping exercise [15]. The program for this event and some presentations from the closing public conference are available online (http://www.equitesante.org/verdas-consortium-workshop-control-and-prevention-of-vector-borne-diseases/).

## Discussion

The VERDAS consortium undertook a far-reaching knowledge synthesis on the control and prevention of vector-borne and other infectious diseases of poverty. Six topics were chosen by an international and multidisciplinary panel of experts. Each scoping review highlighted evidence and implications for public health practice, as well as research needs. The final stage involved collaborative consultation with stakeholders to set priorities among all the research needs identified. This project was an opportunity to present a broad synthesis of current evidence and a list of research priorities to be considered in public health policy and practice, as well as in future research initiatives and donor agendas. The purpose of the present paper is to present the full approach of the VERDAS consortium, to raise some methodological points for consideration, and finally to offer some reflections and lessons learned for future similar consortia.

### Methodological considerations

As mentioned earlier, as we were conducting our project, we began wondering about the fine line between scoping reviews and systematic mixed-method reviews. Scoping reviews are used to map key concepts rapidly and identify research gaps. They can incorporate all study designs, and they generate diverse types of findings, addressing research questions that go beyond intervention effectiveness only. Scoping reviews tend to be non-systematic in nature and to focus on breadth of coverage of the literature rather than on depth of coverage [16]. It is not uncommon for scoping reviews to contain data from both qualitative and quantitative studies, as well as non-research materials, such as commentaries or informal reports of professional meetings [17]. Usually, scoping reviews do not provide in-depth analysis, focusing instead on mapping the available evidence on a broad topic.

Systematic reviews are used to identify and usually evaluate evidence on a particular clinical question [18]. The dominant approach in systematic reviews was, for a long time, the meta-analysis of randomized controlled trials (RCTs), conceptualized as the “gold standard” for synthesizing evidence of effectiveness. Indeed, this type of review is so typical that it has virtually become synonymous with systematic reviews for most researchers, leading to a common misconception of the nature of systematic reviews [19, 20]. The goal of a meta-analysis of RCTs is to produce an overall pooled estimate effect of one specific intervention (e.g. a new vaccine) on one specific health outcome (e.g. dengue). It focuses on research questions such as “What interventions work?” Thus, one of the major ways in which it differs from scoping reviews concerns quality assessment. [5] Systematic reviews are required to assess the quality of the evidence presented [4], which has led to exclusion criteria being applied in numerous meta-analyses. In contrast, the matter of quality assessment is still under debate in relation to scoping reviews; it is rarely done, under the rationale that, by nature, scoping reviews include all relevant studies retrieved in databases, without exclusions based on study design or quality [6]. In our approach, we decided to include qualitative assessment of the studies as a tool to inform readers on the availability of strong evidence. It was applied during data extraction, and as such, there was never any intention to exclude studies based on quality assessment. Because of this, we were sometimes able to highlight a lack of strong evidence despite the availability of several studies.

More importantly, meta-analyses are not the only options in the developing field of systematic reviews, such as systematic mixed-methods reviews, which include both qualitative and quantitative evidence [21]. As this field is still emerging, there is not yet any real consensus on how to conduct this type of review and how to integrate both types of data into one final synthesis [22]. The mixed-methods approach to systematic reviews is a process whereby either a) comprehensive syntheses of two or more types of data are conducted separately and then aggregated into a final combined synthesis (segregated approach), or b) qualitative and quantitative data are combined and synthesized into a single primary synthesis (integrated approach) [23].

It must be noted that scoping reviews are mostly exploratory, undertaken because of time and resource constraints, and used as a preliminary evaluation of the possibility of doing a systematic review. However, scoping reviews should never be considered as “easy”, “rapid”, or “cheap” systematic reviews. In a scoping review, key concepts are **mapped** to understand the availability of literature [5]. In a systematic review, studies’ findings are **interpreted**.

The authors of the present paper consulted experts in review methodologies, and given that a) our approach used a systematic procedural protocol, which supported the reproducibility of results, and b) it provided an analysis of evidence found, we concluded that we might use the term “systematic mixed-method review”. However, in an open discussion with the consortium members, we observed that researchers with medical backgrounds preferred not to use the term “systematic”, given the absence of meta-analysis in our reviews. It may be that the confusion around what specifically constitutes a ‘systematic review’ may be stronger in the biomedical field, where researchers are more exposed to and rely frequently on meta-analysis.

We retained the “scoping” terminology, even though we are aware that we may have crossed the slim methodological border with systematic mixed-method reviews several times, given that the end goal was completely in line with the scoping reviews framework.

### Reflections and lessons learned

We took the opportunity of the final workshop to conduct a short reflexive brainstorming session on the experience of the VERDAS consortium. Here we present a few suggestions for research groups that may be interested in reproducing our approach.

1. *Coordination is important*

Having a research coordinator was essential to deal with both scientific and administrative requirements for this type of international collaboration. This person was a key resource to provide similar detailed protocols for each team, track each team’s progress, and most importantly, strive to ensure a certain consistency among the teams.

2- *Start with a workshop*

Due to budget limitations, we could hold only one workshop with scoping leaders. From the outset, we planned it for the end of the project, to conduct the concept mapping exercise with decision-makers. However, all agreed a launch workshop would have been beneficial on several points. First, it would have helped align everyone’s expectations, as we noticed only in the last stage of the project that contributors did not all have quite the same vision of the content of reviews, and it was very difficult to reorient the content of a review when the draft of a manuscript was already well advanced. Second, a launch workshop would have reinforced the sense of community and networking, as some teams revealed in the brainstorming that they had felt somewhat isolated in this work despite the coordinator’s assistance. Third, it would have been an opportunity to provide training in bibliographic research methods. Most of the researchers and assistants thought doing literature reviews was very intuitive, as they were accustomed to navigating scientific databases every day; but in fact, performing a constructed valid search strategy is complex. Finally, the workshop would have allowed all scoping leaders to be equally involved in the first steps. Given that we had similar key concepts across the consortium, we decided to standardize keywords. However, some teams were delayed in starting their scoping reviews and paid less attention to the related communication. By the time they started their own search strategy, it was no longer possible to integrate their comments, as other teams had already completed the search strategy, and as such, the later teams may have felt excluded.

3- *A librarian is essential*

The involvement of a specialized librarian was essential in building the search strategy. Without it, major errors would certainly have been made.

4- *The workload should not be underestimated*

Most researchers acknowledged that they had not anticipated such a significant investment of time and resources, and said they might think twice before committing themselves again to such work. They were divided on the utility of tools such as TIDieR and ASTAIRE for data extraction. These tools were originally intended to facilitate the work, but some researchers felt they were inappropriate and created more work than necessary. Others found them very useful for a comprehensive data extraction grid.

## Conclusions

In this paper we have presented in detail the approach we used for the VERDAS consortium and discussed certain methodological issues, specifically regarding the fine line between scoping and systematic mixed-method reviews. We believe this rigorous approach to knowledge synthesis should be considered in future research initiatives and donor agendas.

## Additional files

Additional file 1: Multiligual abstract in the five official working languages of the United Nations. (PDF 713 kb)

Additional file 2: eDelphi topics submitted after round 1. (DOCX 16 kb)

Additional file 3: Scoping Review Template. (DOCX 21 kb)

## Abbreviations

ASTAIRE: Analysis of the transferability and support to adaptation of health promotion interventions
LMICs: Low- and middle-income countries
MMAT: Mixed method appraisal tool
TIDiER: Template for intervention description and replication
VBDs: Vector-borne diseases
