# Supplementary material for: Approach to identifying research gaps on vector-borne and other infectious diseases of poverty in urban settings: scoping review protocol from the VERDAS consortium and reflections on the project’s implementation
Source: Infect Dis Poverty. 2018 Sep 3;7:98. doi: 10.1186/s40249-018-0479-3 (PMC6120063; doi:10.1186/s40249-018-0479-3)
Supplement: Supplementary file 1 — Multiligual abstract in the five official working languages of the United Nations. (PDF 713 kb) [file 40249_2018_479_MOESM1_ESM.pdf]

Translation of the abstract into the five official working languages of the United Nations

تدابير التعرف على الثغرات البحثية في الأمراض المتنقلة وغيرها من أمراض الفقر المعدية في البيئات الحضرية: تقييم بروتوكول المراجعة من جمعية "فيرداس" وتداعياته على تنفيذ المشروع

ستيفاني ديقروت، كلارا بيرموديز تامايو وفاليري ريدل

الملخص
خلفية الموضوع: تقدم هذه الورقة المنهج العام الذي تتبعه "جمعية المراجعات التقييمية للأمراض المتنقلة "(فيرداس) استجابة للنداء الصادر من وحدة الأمراض المنقولة بالنواقل والبيئة والمجتمع التابعة للبرنامج الخاص للبحث والتدريب في الأمراض الاستوائية برعاية منظمة الصحة العالمية، يهدف هذا المشروع إلى تكوين معرفة واسعة و تحديد الثغرات المعرفية المتعلقة بالتحكم والحد من الأمراض التي تنتقل بالنواقل في البيئات الحضرية.
الأساليب: يتكون التحالف من 14 باحث , و 13 مساعد بحث, ومنسق ابحاث من سبعة مؤسسات في كندا ,كولومبيا ,البرازيل ,فرنسا ,اسبانيا ,و بوركينا فاسو. تم تطوير بروتوكول من ست خطوات للمراجعات التقييمية التي ينفذها التحالف بناءً على الإطار الذي تم تطويره من قبل أركسي وأومالي وتم تحسينه من قبل لوفاك وآخرين، في الخطوة الأولي , تم التعرف علي ستة مواضيع خلال استشارة عالمية بطريقة "دلفي". وفي خلال الخطوات الأربع التالية، تم تنفيذ المراجعات التقييمية. وكانت الخطوة السادسة هي إقامة ورشة "فيرداس" في كولومبيا في مارس 2017.
المناقشة: في هذه المقالة ، نناقش عدة قضايا منهجية يتم مواجهتها ونشارك آراءنا حول هذا العمل. حيث نعتقد أن هذا البروتوكول يقدم مثالاً قوياً لعملية شاملة و محكمة لتوليف معرفة واسعة لأي من المواضيع المطروحة، و ينبغي أن يتم وضعه في الاعتبار للمبادرات البحثية المستقبلية و أجندات المانحين في العديد من المجالات لتسليط الضوء على احتياجات البحث بشكل علمي.

Translated from English version into Arabic by Madawi Alajmi, proofread by Bishoy ISAAC, through

TRANSLATORS
WITHOUT BORDERS

界定城市环境中媒传疾病和其他贫困所致传染病相关研究空白的方法：媒传疾病勘域综述团队的综述方案和项目实施反思

Stéphanie Degroote, Clara Bermudez-Tamayo and Valéry Ridde

摘要
引言：本文介绍了媒传疾病勘域综述（VERDAS）团队为响应世界卫生组织热带病培训研究特别规划署的病媒、环境和社会办公室提出的号召而采取的一系列措施。该项目旨在综合分析城市环境中媒传疾病防治的相关研究情况，并确定其研究空白点。
方法：该团队由 14 名研究人员、13 名助理和 1 名研究协调员组成，他们来自加拿大、哥伦比亚、巴西、法国、西班牙和布基纳法索的 7 个不同机构。根据 Arksey 和 O'Malley 开发并由 Levac 等人改进的框架，我们为本次勘域综述制定了一个六步方案。第一步通过国际 e 德尔菲咨询方法确定 6 个主题。在接下来的 4 个阶段中，撰写勘域综述。最后于 2017 年 3 月在哥伦比亚举行 VERDAS 研讨会。
讨论：在本文中，我们将讨论遇到的几个方法学问题，并分享对这项工作的反思。我们认为，该研究框架为任何特定主题进行广泛的知识综合提供了详尽而严谨的范例，应考虑将其用于未来多个领域的研究计划，纳入捐助者议程，从而科学地强调研究需求。

Translated from English version into Chinese by Jin Chen, edited by Pin Yang

Approche pour identifier les lacunes de la recherche sur les maladies à transmission vectorielle et autres maladies infectieuses de la pauvreté en milieu urbain: protocole d'examen de la portée du consortium VERDAS et réflexions sur la mise en œuvre du projet

Stéphanie Degroote, Clara Bermudez-Tamayo et Valéry Ridde

Résumé

Historique: Cet article présente l'approche globale entreprise par le consortium «VERDAS» en réponse à l'appel lancé par l'unité Vecteurs, Environnement et Société du Programme spécial de recherche et de formation sur les maladies tropicales organisé par l'Organisation Mondiale de la Santé. L'objectif du projet était d'entreprendre une vaste synthèse des connaissances et d'identifier les lacunes en matière des connaissances concernant le contrôle et la prévention des maladies à transmission vectorielle en milieu urbain.

Méthodes: Le consortium comprend 14 chercheurs, 13 assistants de recherche et un coordinateur de recherche issus de sept institutions différentes au Canada, en Colombie, au Brésil, en France, en Espagne et au Burkina Faso. Un protocole en six étapes a été élaboré pour les examens de la portée effectués par le consortium, sur la base du cadre élaboré par Arksey et O'Malley et amélioré par Levac et al. Au cours de la première étape, six thèmes ont été identifiés lors d'une consultation internationale eDelphi. Dans les quatre étapes suivantes, les examens de la portée ont été effectués. La sixième étape a été l'atelier VERDAS, qui s'est tenu en Colombie en mars 2017.

Discussion: Dans cet article, nous discutons de plusieurs problèmes méthodologiques rencontrés et partageons nos réflexions sur ce travail. Nous croyons que ce protocole fournit un exemple solide de processus exhaustif et rigoureux pour effectuer une synthèse des connaissances sur un sujet donné et qu'il devrait être pris en compte pour les initiatives de recherche à venir et les programmes des donateurs dans plusieurs domaines pour mettre scientifiquement en évidence les besoins de recherche.

Translated from English version into French by Callixte Nizigama, proofread by Sophie N, through

Подход к определению пробелов в исследовании трансмиссивных и других инфекционных болезней нищеты в городских условиях: обзор протокола исследования от консорциума VERDAS и замечания по реализации проекта

Стэфани Дегрут, Клара Бермудес-Тамайо и Валери Ридд

Краткое описание

Общие сведения: В данном докладе представлен общий подход консорциума «VEctor boRne DiseAses Scoping reviews» (VERDAS) (Регламенты контрольных процедур по трансмиссивным заболеваниям) в ответ на запрос подразделения «Векторы, окружающая среда и общество» Специальной программы по научным исследованиям и подготовке специалистов в области тропических болезней Всемирной Организации Здравоохранения. Цель проекта состоит в проведении масштабного синтеза знаний и определении пробелов в данных, касающихся контроля и предотвращения распространения трансмиссивных заболеваний в городских условиях.
Методы: Консорциум состоит из 14 научных сотрудников, 13 их ассистентов и одного координатора исследования из семи разных институтов в Канаде, Колумбии, Бразилии, Франции, Испании и Буркина-Фасо. Для предварительного анализа консорциумом был разработан шестиэтапный протокол на основе концепции, разработанной Аркси и О'Мэлли и усовершенствованной Левак и др. На первом этапе с помощью международной консультации eDelphi были определены шесть тем. На следующих четырех этапах был проведен предварительный анализ. На шестом этапе в марте 2017 г. в Колумбии была проведена конференция VERDAS.
Обсуждение: В данной статье обсуждаются несколько возникших методологических вопросов и приводятся наши размышления по этой работе. Мы считаем, что в данном протоколе приведен яркий пример обстоятельного и тщательного процесса проведения масштабного синтеза знаний по любой заданной теме, который следует принять во внимание в будущих исследовательских проектах и донорской повестке дня в разных областях для акцентирования исследовательских потребностей на научной основе.

Translated from English version into Russian by Tatiana, proofread by Galina Dmitrieva, through

TRANSLATORS WITHOUT BORDERS

Enfoque para la identificación de brechas de investigación sobre enfermedades transmitidas por vectores y otras enfermedades infecciosas que afectan a personas en situación de pobreza en entornos urbanos: protocolo de estudio de alcance del consorcio VERDAS y reflexiones sobre la aplicación del proyecto

Stéphanie Degroote, Clara Bermudez-Tamayo y Valéry Ridde

Resumen
Antecedentes: Este trabajo presenta el enfoque general tomado por el consorcio VERDAS (Estudios de alcance de enfermedades transmitidas por vectores o "VEctor boRne DiseAses Scoping reviews") como respuesta a un llamamiento de la unidad de Vectores, Ambiente y Sociedad del Programa Especial de Investigaciones y Enseñanzas sobre Enfermedades Tropicales de la Organización Mundial de la Salud. El objetivo del proyecto era desarrollar una síntesis de conocimiento amplio e identificar brechas de conocimiento sobre el control y la prevención de enfermedades transmitidas por vectores en entornos urbanos.

Métodos: El consorcio se compone de 14 investigadores, 13 asistentes de investigación y un coordinador de investigación que provienen de siete instituciones diferentes de Canadá, Colombia, Brasil, España, Francia y Burkina Faso. Se concibió un protocolo de seis pasos para los estudios de alcance llevados a cabo por el consorcio, con base en el marco desarrollado por Arksey y O'Malley, y mejorado por Levac et al. En el primer paso, se identificaron seis temas a partir de una consultoría internacional de eDelphi. En los cuatro pasos siguientes, se llevaron a cabo los estudios de alcance. El sexto paso consistió en el taller de VERDAS celebrado en Colombia en marzo de 2017.

Discusión: En este artículo se discuten numerosas cuestiones de metodología que se afrontaron y compartimos nuestras reflexiones sobre este trabajo. Creemos que este protocolo proporciona un ejemplo sólido de un proceso exhaustivo y riguroso para la realización de síntesis de conocimiento amplio para cualquier tema dado y se debe considerar para iniciativas futuras de investigación y agendas de donantes en campos múltiples a fin de poner de relieve necesidades de investigación de manera científica.

Translated from English version into Spanish by Macarena Belén Pierrot, proofread by María Diehn, through

TRANSLATORS
WITHOUT BORDERS
