# Supplementary material for: Approach to identifying research gaps on vector-borne and other infectious diseases of poverty in urban settings: scoping review protocol from the VERDAS consortium and reflections on the project’s implementation
Source: Infect Dis Poverty. 2018 Sep 3;7:98. doi: 10.1186/s40249-018-0479-3 (PMC6120063; doi:10.1186/s40249-018-0479-3)
Supplement: Supplementary file 2 — eDelphi topics submitted after round 1. (DOCX 16 kb) [file 40249_2018_479_MOESM2_ESM.docx]

**Additional file 2: eDelphi topics submitted after round 1**

Development and implementation in urban areas of low-cost, simple and/or rapid diagnostic technologies for vector-borne and other infectious diseases of poverty

Impact evaluations of different types of interventions in urban settings for vector control : nebulization, breeding places elimination, larvicide use, community-based interventions

Housing, water and sanitation: issues and solutions related to the prevention and control of vector-borne diseases and other infectious diseases of poverty in urban areas

Social mobilization and involvement of urban communities in control interventions against vectors, especially in the elimination of breeding and larvae places

Transmission dynamics, vectorial capacity and coinfections : impacts on the epidemiology of vector-borne diseases in urban areas

Social and cultural determinants of health: inequities in the burden of vector-borne diseases in urban areas

Evaluation of cost-effectiveness and sustainability of integrated vector management in urban settings to prevent vector-borne diseases

Specific needs for the most vulnerable populations (mobile migrant population and internally displaced people) for the prevention and control of vector-borne and other infectious diseases of poverty in urban areas

Epidemiological history of vector-borne and other infectious diseases of poverty in urban areas : identifying hot spots and preventive control approach

Real-time monitoring and epidemic detection in urban settings : evaluation of rapidity and efficiency of containment measures of emerging and re-emerging vector-borne and other infectious diseases of poverty

Control programs and vector ecology: how to apply specific measures according to different contexts in urban settings

Development of effective surveillance systems for vector-borne diseases in urban settings and translating the data into action

Vaccination and awareness campaigns for vector-borne diseases : coverage, access and answers in poor urban communities

Sanitation, hygiene, water, waste and infrastructures management in urban areas : benefits of cross-sectional interventions and effectiveness in vector controls

Social impacts and economic repercussions of vector-borne and other infectious diseases of poverty epidemics in urban settings

Economic dynamics of vector-borne and other infectious diseases of poverty epidemics in urban settings : evaluation of healthcare costs, preventive measures costs and indirect costs

Risk assessment of emergence or re-emergence of vector-borne and other infectious diseases of poverty in urban centers

Mapping insecticide resistance and new insecticides use in cities

Perceptions and acceptability for urban communities : impacts on cost-efficiency, implementation and strategy for sustainable social participation to health interventions for prevention and control of vector-borne and other infectious diseases of poverty

Methodological approaches of urban health interventions for prevention and controls of vector-borne diseases

Coordination, leadership, roles distribution, political willingness : governance in the implementation of actions against vector-borne diseases in urban areas

Vaccination strategies (population and geographical sampling) for future vaccines campaigns (dengue, zika) in urban areas

Healthcare services factors (access, quality…) associated with lethality of vector-borne diseases in urban settings

Challenges and difficulties to produce evidence on implementation and evaluation of vector control strategies in urban areas : review of efficiency and impact indicators

The International Public Health Emergency on Zika : Distribution, control vector, surveillance, intervention, screening, acute and chronic events

Promotion of community-based and inter-sectoral interventions on integrated vector management in urban settings

Population dynamics and vectors distribution: impacts on the epidemiology of vector-borne and other infectious diseases in urban areas

Proximity of early warning systems and response to epidemics in urban settings: risk stratification of transmission of vector-borne and other infectious diseases of poverty at neighbourhood level

Comparative evaluation of cost-efficiency of interventions and tools for vector controls to fit the conditions of poor urban settings

Underreporting in surveillance systems of vector-borne and other infectious diseases of poverty in urban areas : extent of problems and strategy to meet them

Comparison of costs to address an epidemic outbreak versus preventive measures : example for dengue in urban settings

Urbanicity and vector dynamics : impact of highly urbanized settings on emergent or re-emergent vector-borne diseases epidemics

Efficiency of healthcare in urban areas for vector-borne diseases, with dengue as an example

Prevent epidemic outbreaks in urban zones : modelizing and targeting hotspots of transmission for timely vector control interventions

Adaptation to urban environment of vectors and evolution of resistance to chemicals substances in urban areas : cross resistance insecticides-pollutants

Impacts of climate change and urbanization trends on the transmission dynamics of vector-borne and other infectious diseases of poverty in urban and peri-urban communities

Cost estimation of vector control and sanitation measures in urban settings : example of the economic value of Aedes aegypti elimination

The impacts of changing trends in urbanization (over the past 50 years and into the future) on the transmission dynamics of vector-borne and other infectious diseases of poverty

World trade, regional and international travels and transportations : impacts of globalization on distribution and spread of vector-borne and other infectious diseases of poverty in urban areas

Indicators, tools, determinants to assess implementation of research and uptake of research findings to support effective prevention and control of vector-borne and other infectious diseases of poverty in urban areas

How to improve advocacy of government/local authorities to reinforce urban communities awareness in vector-borne diseases prevention and control?

How to prioritize interventions of surveillance, prevention or control of vector-borne and other infectious diseases of poverty depending on epidemiologic and socio-economic status in urban settings

Stamping out urban vectors: focus on scientific innovations in programs to control the spread of vectors

Healthcare services in urban settings for vector-borne and other infectious diseases of poverty : evolution of availability and access to services

Potential threats of pesticides used in agricultural sectors to insecticide resistance of vectors in urban areas of developing countries

Inter-sectoral action and community-oriented services to influence the prevention and control of vector-borne and other infectious diseases of poverty control in urban settings

Heterogeneity in behavior and biodiversity of vectors in urban settings : new tools for monitoring and their potential impact on control interventions

Community-based management of vector-borne diseases in urban settings : actors cartography and therapeutic itinerary

Chronic sequel and long-term impacts of vector-borne diseases : physical health, intellectual development and quality of life in urban settings

Modelling impact of vaccines and vector control scenarios : dengue example

Identification and implication of vectors in vector-borne diseases in urban settings

Vector-borne and other infectious diseases of poverty surveillance and community-based risk communication in urban areas: technological and social innovation in routine public health practice and in outbreak situations

Current state of insecticides for vectors control in urban settings : innovation et methods of use

Efficiency of information flow from the surveillance systems and risk communication programs in urban settings on the adoption of preventive behaviors

Evolution and projection into the future of climatic changes and urban micro-climates on vector-borne diseases dynamics into the cities : vectors and vectorial capacity, pathogenic agents, transmission

Interventions on health promotion and prevention of vector-borne and other infectious diseases of poverty : issues and solutions in urban communities

Global politics of control srategies for vector-borne and other infectious diseases of poverty in urban areas : political, contextual and financial factors needed in order to extend strategies in urban zones

Methodology related to sampling and sample size for vectors population in urban areas

Comparison between new technologies and development of low tech solutions to vector control in urban areas

Vector population genetic structures: influence on vector distribution, adaptation to new habitats, interventions and epidemiology of vector-borne diseases in urban settings

Epidemiologic monitoring of neglected tropical diseases in big cities : specificity et restrictions for better effectiveness

Paradigm change and interaction between civic (education and cultural) and governmental (social trust) : reinforce community actions in conjunction with local public health system to strengthen the fight against vector-borne diseases in cities

Private industries and non-health sectors in vector-borne diseases control in urban zones

Analysis of messages and information sources (social media, social marketing…) in urban settings for the prevention of vector-borne diseases, especially for the young people

Innovative approaches in prevention and fight against vector-borne and other infectious diseases of poverty using new entomologic criterion

Care for vector-borne diseases in urban settings : locales therapy, self-treatment and/or iatrogeny

Efficiency evaluation in urban settings and at population level for vector-borne diseases vaccines available (example malaria) or in development (example dengue)

Behavorial impact of urban communities on vector-borne pathogens resistance to anti-infectious drugs

Impact of climate and environmental changes on genetic variability of pathogenic agents with vectorial transmission in urban zones

Privatisation of healthcare services in urban settings : impacts on access and care of vector-borne and other infectious diseases for the poor

Methods for comparative analysis of gaps in diagnostic and healthcare of vector-borne and other infectious diseases of poverty in urban settings at a regional level

Vector-borne diseases and acute and chronic diseases : issues and solutions for taking into account specific needs in health interventions

Study of blood meals in vectors population according to their habitats (urban vs peri-urban vs rural vs forest vs with important migration dynamics…) : characterisation of hosts and potentials reservoirs of pathogenic agents with infectious risk for human population

Mapping of mixes of vectors in urban and peri-urban settings, by region, and their longitudinal evolutions

Development of biomarkers of the evolution of care for acute and chronic vector-borne diseases

Effectiveness and added value of urban community-based vectorial surveillance (or other animal reservoirs) for targeting interventions in urban settings

Design of bednets: factors to improve their use in uban settings

Acceptability for national regulatory bodies of control gene-drive interventions against vectors in urban areas

Monitoring of institutional reponses in health sector, local governements, population and private sectors when facing epidemic outbreaks of vector-borne diseases in urban areas

History of policies and health interventions programs for prevention and control of vector-borne and other infectious diseases of poverty in urban areas

Minimum data requirements for introducing a new tool for control urban vectors of disease

Finding ways to encourage novel research of vector control useful in urban settings such as reduction of vectoring capacity due to endemic co-infections and genetically altering mosquitos

The contribution of sanitation to climate changes and its association with vector borne diseases in urban areas

Green spaces and animals living there, forest relics, rapid and uncontrolled changes in urban zones : impacts of urban environment on vector-borne and other infectious diseases of poverty

Basic research on pathogen-invertebrate interaction to develop new targets for potential intervention in an urban setting

**Topics first submitted to TDR-WHO**

Vector-borne disease transmission dynamics in urban settings: A review of entomological and epidemiological issues and research

Vector-borne and other infectious diseases of poverty in urban settings: A comparative perspective on the evolution of urbanization trends (with a focus on the past 10-20 years and projections into the future).

Housing, water and sanitation: Issues in and solutions for infectious diseases prevention and control

Implementation research findings on infectious diseases prevention and control in urban settings : Issues and solutions in large scale interventions

Vector-borne diseases surveillance and community-based risk communication in urban areas: technological and social innovation in routine public health practice and in outbreak situations

Social dynamics and governance issues influencing urban vector-borne diseases control with an emphasis on inter-sectoral planning, communication and action, and community-oriented services

Population dynamics and social determinants of health interactions with vector-borne and other infectious diseases prevention and control in urban areas

Surveillance and control programs against urban vectors: review on scientific innovations and cost efficiency in public health strategies

Interventions on health promotion and vector-borne diseases : issues and solutions of preventive education in urban communities

Vector-borne diseases and chronic diseases : issues and solutions of encountering specific needs in health interventions

Methodology related to the sampling and sample size for vectors population in urban areas
