# Supplementary material for: Approach to identifying research gaps on vector-borne and other infectious diseases of poverty in urban settings: scoping review protocol from the VERDAS consortium and reflections on the project’s implementation
Source: Infect Dis Poverty. 2018 Sep 3;7:98. doi: 10.1186/s40249-018-0479-3 (PMC6120063; doi:10.1186/s40249-018-0479-3)
Supplement: Supplementary file 3 — Scoping Review Template. (DOCX 21 kb) [file 40249_2018_479_MOESM3_ESM.docx]

**Scoping Review Template**

**Title:** The title should be informative and give a clear indication of the topic of the scoping review. The title should also include the phrase “…: a scoping review” to allow easy identification of the type of document for potential readers.

**Authors:** Based on the tables of contribution, list all people that should be include as principal, middle and last authors. All people listed here must have made substantial contribution to the work.

**Abstract:** Should be written at last. In 300 words maximum, construct your abstract with the following sections: Background; Aim; Design/methods; Results; Conclusions.

**Introduction: 350 words**

1- Background section: it should be comprehensive and cover all the main elements of the topic under review. Due to scoping reviews being essentially exploratory, it is not expected that the background covers the extant knowledge in the area under review. The background should also detail any definitions important to the topic of interest.

The background section must include an indication of whether or not there are existing reviews, systematic reviews, research syntheses, and/or primary research papers available on the topic, hence supporting the rationale to conduct the scoping review.

The background section should conclude with a statement that a preliminary search for existing scoping reviews on the topic has been conducted. If there is an existing scoping review available on the topic, a justification that specifies how the proposed review will differ from that already conducted and identified should be detailed.

2- Scoping objective: The reason for undertaking the scoping review should be clearly stated together with what the scoping review is intended to inform. The objective of the scoping review should indicate what the scoping review project is trying to achieve. The objective may be broad and will guide the scope of the enquiry.

**Methods: 500 words**

1- Scoping topic definition: Few lines will be dedicated at the beginning of this section to the Delphi process used to select all six topics of scoping. This section will be prepared by Stéphanie and send it to you.

2- Search strategy: use your search strategy form to write this section. This section should explicit exactly how the databases search was done. The reader of the article should be able to perform the exact same research as you and having the same results.

3- Inclusion / Exclusion criteria: State your inclusion / exclusion criteria and justify them.

4- Data extraction: Explain how you performed the data extraction (validation round(s) for the grid for example). Present succinctly the tool used: MMAT, TiDIER, ASTAIRE.

**Results: 2000 words**

1- Description of studies included and sources: The results section must tart by identifying how many studies were identified and selected. There should be a narrative description of the search decision process accompanied by the search decision Prisma Chart. The Prisma chart should clearly detail the review decision process, indicating the results from the search, removal of duplicate citations, study selection, full retrieval and additions from a third search.

This section should also include an overall description of the included sources. A narrative summary should logically provide details to support the inclusion of each source (paper, study, report, etc.) in the scoping review.

2- MMAT Graphic: you should start your manuscript with a brief description of the quality of studies included and then insert the MMAT graphic.

3- Graphic presentation of the data: The usual way to present results is to use a copy of the data extraction grid completed, and then summarize it in order to present key findings in a logical, descriptive and readable way. But, when appropriate, the results of a scoping review may be presented as a map of the data extracted from the included papers in a diagrammatic or tabular form.

4-Description of the data: Describe in logic order the finding of your scoping work, in a descriptive format that aligns with the objective and scope of the review

**Discussion: 1000 words**

1- Discussion of the results: This section should discuss the results of the review as well as any limitations of the sources included in the scoping review. Results should be discussed in the context of current literature, practice and policy. For example, scoping reviews are subject to the same limitations as any other type of review such as: relevant sources of information may be omitted and the review is dependent on information being available.

2- Implications for future research: This section should include clear, specific recommendations for future research based on gaps in knowledge identified from the results of the review. You may make comments about the needs for future conduct of primary research in the area of interest. In this section we strongly encourage you to include a box of top priority research needs according to your results

Knowledge gaps and priority needs Implications for public health

for future research: policy and/or practice:

- . - .
- . - .
- . - .
- . - .

3- Implications for public health policy and/or practice: This section should include clear results from the scoping review that can be used to inform practice. It may also be possible to develop recommendations for practice / policy based on the results of the scoping review.

**Conclusions: 150 words**

This section is an overall conclusion based on the results and should highlight top priority research needs or policy recommendations. The conclusions drawn should match the review objective.

**Ethic statement:** A statement which either declares the absence of any conflicts of interest or which describes a specified or potential conflict of interest should be made by the reviewers in this section.

**Acknowledgments:** Any acknowledgments should be made in this section, such as sources of external funding or the contribution of colleagues or institutions. People who contributed in the scoping work but not in such substantially manner that they have not been included as co-authors should be acknowledge here.

**References:** List of all references cited in the manuscript. Please use Zotero to insert your references. The first time you will insert a reference in your text Zotero will ask you the format desire. We will all use the format called: “ieee”. All references will be insert in the text in this way: [1]
